# Screening for Depression in the General Population with the Center for Epidemiologic Studies Depression (CES-D): A Systematic Review with Meta-Analysis

**DOI:** 10.1371/journal.pone.0155431

**Published:** 2016-05-16

**Authors:** Gemma Vilagut, Carlos G. Forero, Gabriela Barbaglia, Jordi Alonso

**Affiliations:** 1 Health Services Research Group, IMIM (Institut Hospital del Mar d’Investigacions Mèdiques), Barcelona, Spain; 2 CIBER Epidemiología y Salud Pública (CIBERESP), Madrid, Spain; 3 Department of Experimental and Health Sciences (DCEXS), Universitat Pompeu Fabra (UPF), Barcelona, Spain; 4 Department of Assessment, Agència de Qualitat I Avaluació Sanitàries de Catalunya (AQuAS), Barcelona, Spain; Tilburg University, NETHERLANDS

## Abstract

**Objective:**

We aimed to collect and meta-analyse the existing evidence regarding the performance of the Center for Epidemiologic Studies Depression (CES-D) for detecting depression in general population and primary care settings.

**Method:**

Systematic literature search in PubMed and PsychINFO. Eligible studies were: a) validation studies of screening questionnaires with information on the accuracy of the CES-D; b) samples from general populations or primary care settings; c) standardized diagnostic interviews following standard classification systems used as gold standard; and d) English or Spanish language of publication. Pooled sensitivity, specificity, likelihood ratios and diagnostic odds ratio were estimated for several cut-off points using bivariate mixed effects models for each threshold. The summary receiver operating characteristic curve was estimated with Rutter and Gatsonis mixed effects models; area under the curve was calculated. Quality of the studies was assessed with the QUADAS tool. Causes of heterogeneity were evaluated with the Rutter and Gatsonis mixed effects model including each covariate at a time.

**Results:**

28 studies (10,617 participants) met eligibility criteria. The median prevalence of Major Depression was 8.8% (IQ range from 3.8% to 12.6%). The overall area under the curve was 0.87. At the cut-off 16, sensitivity was 0.87 (95% CI: 0.82–0.92), specificity 0.70 (95% CI: 0.65–0.75), and DOR 16.2 (95% CI: 10.49–25.10). Better trade-offs between sensitivity and specificity were observed (Sensitivity = 0.83, Specificity = 0.78, diagnostic odds ratio = 16.64) for cut-off 20. None of the variables assessed as possible sources of heterogeneity was found to be statistically significant.

**Conclusion:**

The CES-D has acceptable screening accuracy in the general population or primary care settings, but it should not be used as an isolated diagnostic measure of depression. Depending on the test objectives, the cut-off 20 may be more adequate than the value of 16, which is typically recommended.

## Introduction

Major Depression ranks amongst the most burdensome health conditions, both at individual and population levels [1–3]. It is the most frequent mood disorder, with a lifetime prevalence that has been reported to range from 7% to 21% [4]. It is also associated with a substantial functional impairment, diminished quality of life, increased burden, both for patients and caregivers, as well as with a higher risk of mortality [4–6]. That notwithstanding, it is estimated that about 50% of depressed patients are incorrectly identified by general practitioners in routine unassisted diagnosis of depression [7], and that only a limited proportion of cases receive adequate treatment [8;9].

Given these figures, systematic screening has been advocated as a means for improving detection, treatment and outcomes of depression, and also to facilitate follow-up of patients’ progress [10;11]. Depression screening would also be important in primary care and general population settings for monitoring disease prevalence [12], as well as for targeting interventions at either individual or group level. Therefore, there is an increasing need for evidence about the accuracy (the ability to discriminate between people with the disorder and those without it) of different assessment methods.

A number of excellent exhaustive diagnostic instruments exist for depression diagnosis (i.e. Diagnostic Interview Schedule (DIS); Composite International Diagnostic Interview (CIDI); Schedules for Clinical Assessment in Neuropsychiatry (SCAN); Mini-International Neuropsychiatric Interview (MINI); Structured Clinical Interview for DSM Disorder (SCID) [12]), but they are generally unfeasible in population-based surveys. Using such instruments is also unattainable in clinical applications with stringent time-demands, and thus outside specialized mental health care services. In contrast, brief, self-reported scales are economical, can be readily applied requiring neither extensive training nor time, and have been found to be sensitive to changes over time. Several short scales have been developed to ascertain the presence of depression in general population surveys and primary care samples [13]. Arguably, the Center for Epidemiologic Studies Depression Scale (CES-D) [14] is one of the most widespread brief scales for assessing depression. Originally devised for screening and research in general population epidemiological studies and primary care, the CES-D has also been extensively used in other settings, as a measure of depressive symptomatology among individuals with specific chronic conditions [15;16], and even as a stand-alone diagnostic measure of depression [17–19].

A simple search in bibliographic databases retrieves thousands of population-based and clinical studies using the CES-D in the last ten years. A number of these studies have evaluated the diagnostic accuracy of the CES-D to detect major depression at the general population and primary care levels. In spite of its prominence, no work has been done to date to integrate the results on its performance through meta-analysis. A meta-analytic approach would be important for the CES-D, since it would provide precise and generalizable evidence about the performance of the CES-D and the interpretation of its results, and to establish whether and how associated findings vary by particular subgroups [20;21].

### Aims of the study

The aim of our study was to collect and analyse all the existing evidence regarding the performance of the CES-D scale to detect major depression in general populations and primary care. This was done through: a) a systematic review to identify the studies evaluating the accuracy (or criterion-related validity) of the CES-D for major depressive disorder in the general population or primary care; and b) a meta-analysis of the available literature.

## Material and Methods

### The Center for Epidemiologic Studies Depression (CES-D) Scale

The CES-D scale is a short self-report scale designed to measure the current level of depressive symptomatology in the general population [14]. It contains 20 items about symptoms that occurred in the week prior to the interview with response options from 0 to 3 that refer to frequency of the symptoms. The score ranges between 0 (best possible) to 60 (worst) and the cut-off point that has been typically recommended for depression caseness is 16 [22]. Individuals with a score of 16 or more must have had either at least 6 of the 20 symptoms in the CES-D with persistence for most of the previous week, or a majority of the symptoms on the scale for shorter periods of time. CES-D literacy level has been defined as easy, and it takes between 2 and 5 minutes to complete.

### Search strategy

We searched for articles in two databases, PubMed and PsychINFO (EBSCOHost), for English and Spanish language journal articles published from January 1st, 1977 through June 15^th^, 2015 (date last searched: June 25^th^, 2015). Search terms included three components that had to be fulfilled. The first component referred to the assessment of mental disorders and included the MeSH terms “mental disorders”, or “anxiety disorders”, or “anxiety”, or “depressive disorder”, combined with the terms “depressive* or depression* or anxiety* or mental disorder*” in title or abstract. The second component dealt with screening instruments and included the MeSH terms “Mass screening”, or “screen*”, and the names of common screening and diagnostic instruments, including “CES-D”, or “CESD”, or “Center for Epidemiologic Studies Depression Scale”, in title or abstract. Finally, the third component had to do with the assessment of the diagnostic accuracy of the instruments including terms related with validation studies, like ROC analysis or sensitivity and specificity. The search strategy applied in PubMed is available in S1 Table.

### Selection of eligible studies

This study was part of a larger project that aimed to examine test characteristics of frequently used screening instruments for common mental disorders, with special interest in the CES-D and the General Health Questionnaire (GHQ). Even though the search strategy was open to all the screening instruments available for mood or anxiety disorders, in this particular work we exclusively focus on the studies identified that used the CES-D scale. Studies were eligible if they met all of the following inclusion criteria: a) they were validation studies of screening questionnaires that reported information on the accuracy of the CES-D instrument for the detection of major depression; b) the sample was taken from the general population or from primary care; c) the diagnosis of mental disorders was done using standardized diagnostic interviews based on either the International Classification of Diseases (ICD) or the Diagnostic and Statistical Manual of Mental Disorders (DSM) classification systems; and d) the language of publication was either English or Spanish. Studies were excluded if: a) the samples were obtained from hospital settings or psychiatric services, or patients suffered specific disorders or they were in specific situations, such as pregnancy; or b) not enough information was provided to obtain 2x2 tables for a specific cut-off point. Assessment of eligibility of studies across all steps of the systematic review was conducted by pairs of independent reviewers (n = 6). Discordances between reviewers regarding eligibility status were solved by consensus in a personal meeting between the reviewers and three investigators (JA, GV and CGF). Data extraction for the studies finally selected was also conducted by pairs of reviewers using an excel form specifically created for this review. Each reviewer extracted data independently and inconsistencies were resolved by a third reviewer.

### Quality assessment

The assessment of the quality of the studies was based on the QUADAS tool [23], following Cochrane review guidelines for the evaluation of diagnostic studies [24]. The QUADAS consists of a set of 14 items, phrased as questions with three response options each (yes, no, unclear), that assess the appropriateness of different aspects of a study, such as the selection of participants, the administration of the test and the gold standard, or whether the data gathering process and results are accurately and comprehensively reported. Out of the 14 original QUADAS items, we assessed the 10 items that relate to bias, as recommended by the authors [24]. One of the items, *“Were the same clinical data available when test results were interpreted as would be available”*, was not assessed because it was irrelevant in the context of this review where the interpretation of the index test is fully automated [23]. Additionally, we included an item asking whether the diagnostic accuracy results were provided for a pre-specified cut-off point or if the selection of the cut-off point was driven by the results of the study. The inclusion was based on available evidence showing that selecting a cut-off that optimizes the diagnostic accuracy in a specific study may lead to overoptimistic estimates of the test performance [20]. The tool is evaluated at the item level and it does not incorporate an overall quality score.

### Statistical analyses

If not provided in the article, the contingency table of true positives, false negatives, true negatives and false positives was constructed for each cut-off point assessed based on the available information, usually sensitivity, specificity and prevalence of the disorder according to the gold standard.

The suggested cut-point for the scale is 16, but many studies presented accuracy results of the CES-D using other cut-points, since they aimed to evaluate and compare their performance and select the optimal value in that specific population. However, for the assessment of overall performance of the scale using information from all the 28 studies, we selected results for only one cut-off point per study, so that each study contributes only one estimate of sensitivity and specificity as required by the statistical methods applied[21]. We chose the cut-off point of 16 whenever possible, as this is the value usually recommended for the detection of depression with the CES-D. Notwithstanding, when a study did not report diagnostic accuracy results for cut-off 16, we used the cut-off point reported in that particular study. When more than one cut-off point was reported in an article, and in order to avoid multiple testing effects, we selected the cut-off with the best diagnostic accuracy within the study. We obtained the coupled forest plot reporting the raw data consisting of the 2x2 sensitivity and specificity table from each study, as well as the estimated sensitivity (SN; the proportion of true cases correctly classified by the cut-off point) and specificity (SP; the proportion of true non-cases correctly classified) for detection of depression of each of the studies, together with 95% confidence intervals. In the context of meta-analysis, when a variety of sensitivity and specificity values for a given test are available from several independent studies depending on the cut-off point, the summary receiver operating characteristic (SROC) has been proposed as a way to assess diagnostic data [25]. The SROC curve considers both sensitivity and specificity and the relationship between them, taking into account that not all studies used the same cut-off. It is assumed that different values of sensitivity and specificity apply if the cut-off point defining a positive test result varies from study to study, everything else being equal. Several procedures have been proposed to estimate the SROC curve from a set of independent studies [25–27]. Here, the Rutter and Gatsonis mixed effects model [26] was fitted to estimate the SROC curve, and the sensitivity and specificity of each study, weighted by study size were plotted in the ROC space. The area under the curve (AUC) for the fitted SROC was computed from the estimated diagnostic odds ratio (DOR) following the method described by Walter [28]. Also, for the subsample of studies that provided diagnostic accuracy results for the cut-off point 16 (n = 22), we estimated a bivariate meta-regression [27], which allowed us to obtain pooled estimates of a range of summary performance measures of the test’s ability to detect the presence of a disease for a given cut-off point. Specifically, the summary measures obtained were: a) specificity and sensitivity, and their corresponding 95% confidence intervals; b) the positive likelihood ratio (LR+) that described how many times more likely positive test results were in the diseased group compared to the non-diseased group; c) the negative likelihood ratio (LR-), describing how many times less likely negative test results were in the diseased group compared to the non-diseased group; and d) the DOR, that summarizes the diagnostic accuracy of the test as a single number describing how many times higher the odds are of obtaining a positive test result in a diseased rather than in a non-diseased person. Additionally, we evaluated the screening accuracy of other cut-off points that were assessed in a minimum of 6 studies using the same methodology. In this case, a separate bivariate model was estimated for each of the cut-off points, and each study could contribute to one or more of the models depending on what cut-off points it reported [21].

The following variables were assessed as possible sources of heterogeneity: a) the study setting; b) the measure used as the gold standard; c) the version of the instrument (English versus cultural adaptation); d) the age group of the study sample; e) disorder prevalence; and f) specific QUADAS items for which more than 20% of the studies presented problems. Heterogeneity was evaluated with the Rutter and Gatsonis mixed effects models (see above) including each covariate at a time and testing its statistical significance with the likelihood ratio test. Estimates of model parameters were obtained using the METADAS macro [29] implemented in SAS (SAS v9.1.2) [30].

## Results

**Fig 1:**
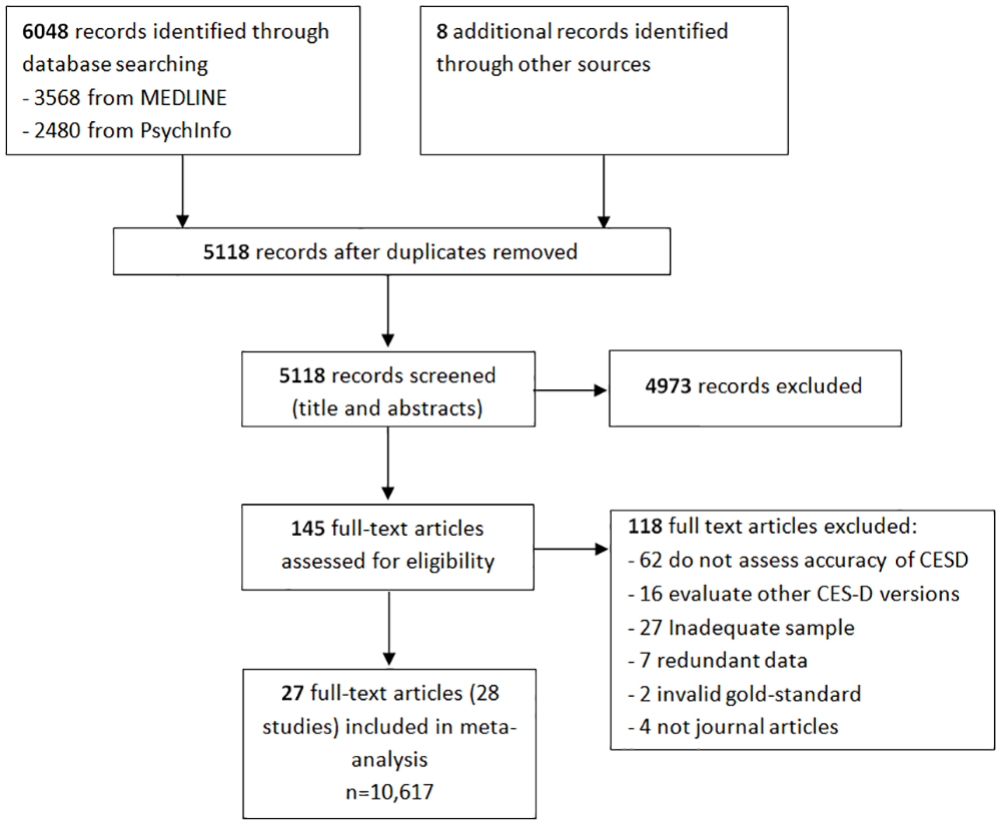
Flow diagram of study selection process.

In our systematic review we identified 5,118 studies. Of them, 4,973 were excluded after title and abstract review (Fig 1). Full text review of the 145 potentially eligible articles was carried out by pairs of reviewers and resulted in 118 articles being excluded. Reasons for exclusion are detailed in Fig 1.

We found 27 articles that met inclusion criteria (n = 10,617 participants) with a total number of 28 studies [31–57] (Table 1). This is because one of the articles [32] assessed the scale properties using independent eligible samples from two different settings (primary care, nursing home), which were included in the review as two separate studies. Twenty-two of the studies presented results for the cut-off point 16, while the other 6 used a different cut-off point, usually claimed to be the optimal one in that specific study. The gold standard used differed across studies, but the most common were: a) the Diagnostic Interview Schedule (DIS) (n = 7 studies); b) the Structured Clinical Interview for DSM (SCID) (n = 6); c) the Composite International Diagnostic Interview (CIDI) (n = 4 studies), and d) the Mini-International Neuropsychiatric Interview (MINI) (n = 4). Studies were conducted in diverse settings: 10 were conducted in primary care, 14 on representative samples from the general population (n = 8) or school samples (n = 6), and the remaining studies (n = 4) were conducted in specific settings for the elderly (e.g. nursing homes, residential homes or community centres). Ten studies used cross-culturally adapted versions of the CES-D questionnaire into other languages, and 3 additional studies specifically indicated that they allowed participants to choose the interview language (English/Spanish or English/Chinese). Eleven studies focused exclusively on older adults and 6 were carried out in adolescents. The median prevalence of Major Depression was 8.8% (IQ range from 3.8% to 12.6%).

**Table 1:** Description of eligible studies for the assessment of diagnostic accuracy of the CES-D (n = 28).

Study	Sample size	Gold Standard	Setting	Age group	Prevalence of depression	% QUADAS items with problems
Beekman, 1997 [31]	487	DIS—Affective/Anxiety, DSM-III	General population	Middle Age/Elderly	2.0%	9%
Blank, 2004 (Primary care) [32]	125	DIS—Mood sections, DSM-IV	Primary care	Middle Age/Elderly	11.0%	9%
Blank, 2004 (Nursing home) [32]	85	DIS—Mood sections, DSM-IV	Residential#	Middle Age/Elderly	9.0%	9%
Camacho, 2009 [33]	390	SCID, DSM-IV	Schools	Adolescent	11.5%	0%
Campo-Arias, 2007 [34]	266	SCID, DSM-IV	General population	Adult	16.5%	0%
Cho, 1993 [35]	2008	DIS—Depression, DSM-III	General population	Adult	3.9%	18%
Christensen, 2011 [36]	326	MINI V6, DSM-IV	General population	Adult	6.9%	9%
Cuijpers, 2008 [37]	243	MINI, DSM-IV/ICD-10	Schools	Adolescent	4.2%	9%
Dozeman, 2011 [38]	277	MINI, DSM-IV	Residential#	Middle Age/Elderly	12.6%	27%
Fechner-Bates S, 1994 [39]	425	SCID, DSM-III-R	Primary care	Adult	12.5%	18%
Garrison, 1991 [40]	332	K-SADS, DSM-III; CGAS	Schools	Adolescent	8.5%	18%
Gerety, 1994 [41]	128	SCID, DSM-III-R	Residential#	Middle Age/Elderly	26.0%	0%
Head, 2013 [42]	274	CIS-R, ICD-10	General Population	Middle Age/Elderly	3.8%	9%
Hendrie HC, 1995 [43]	125	CAMDEX, DSM-III-R	Primary care	Middle Age/Elderly	1.8%	9%
Lewinsohn, 1997 [44]	1005	SADS, DSM-III-R	General population	Middle Age/Elderly	8.0%	18%
Li, 2010 [45]	166	CIDI 2.1	General population	Adult	1.8%	9%
Lyness, 1997 [46]	130	SCID, DSM-III-R	Primary care	Middle Age/Elderly	9.2%	18%
McQuaid, 2000 [47]	213	UM-CIDI, DSM-III-R	Primary care	Adult	23.9%	9%
Papassotiropoulos, 1999 [48]	287	CIDI, DSM-III-R	General population	Middle Age/Elderly	3.5%	18%
Perez-Stable, 1990 [49]	265	DIS, DSM-III	Primary care	Adult	26.4%	27%
Prescott, 1998 [50]	556	DISC, DSM-III-R	Schools	Adolescents	8.5%	9%
Ring, 1991 [51]	48	SCID, DSM-III-R	Primary care	Adult	28.0%	0%
Roberts, 1991 [52]	1704	K-SADS, DSM-III-R	Schools	Adolescent	2.5%	9%
Robison, 2002 [53]	303	CIDI, DSM-IV	Primary care	Middle Age/Elderly	12.0%	0%
Ros, 2011[54]	58	MINI, DSM-IV	Residential#	Middle Age/Elderly	37.9%	9%
Thomas, 2001 [55]	179	DIS, DSM-IV	Primary care	Adult	11.0%	9%
Yang, 2004 [56]	178	K-SADS—Epidemiology	Schools	Adolescent	2.4%	9%
Zich JM, 1990 [57]	34	DIS, DSM-III	Primary care	Adult	5.8%	18%

CAMDEX: Cambridge Mental Disorders of the Elderly Examination; CIDI: Composite International Diagnostic Interview; CIS-R: Revised Clinical Interview Schedule; CGAS: Children’s Global Assessment Scale; DIS: Diagnostic Interview Schedule; DIS: Diagnostic Interview Schedule for Children; DSM: Diagnostic and statistical manual; K-SADS: Schedule for Affective Disorders and Schizophrenia for Children; MINI: Mini-International Neuropsychiatric Interview; SADS: Schedule for Affective Disorders and Schizophrenia; SCID: Structured Clinical Interview.

^#^ Residential: Nursing homes, Residential home or Community centers

### Quality of the studies

With regard to the methodological quality of the studies, the QUADAS item that presented higher number of problems was “partial verification”, where half of the studies (n = 14) were positive, meaning that the gold standard was not administered to the whole sample or a random selection of it, and usually the results of the index test influenced the decision to perform the reference standard. However, in 8 out of the 14 studies with partial verification, the reference standard was administered to all respondents over a specific cut-off point in the CES-D and a random proportion of the rest. Importantly, in all those studies, the analyses were adequately corrected for the oversampling of positive test respondents, mainly by weighting the participants by the inverse of their probability of selection. Under-reporting of methods was substantial: 25% of the studies did not provide information about withdrawals in the CES-D scale or the reference standard. Half of the studies supplied no information regarding whether the reference standard results were blinded to the CES-D and nearly 40% did not report about the time between administrations of the two tests. Details on blinding of the CES-D results to the gold standard were not reported in 11% of the studies. Four studies reported sensitivity and specificity exclusively based on optimal cut-off points determined by post-hoc receiver-operating curve (ROC) analyses (see S1 Fig).

### Accuracy results

**Fig 2:**
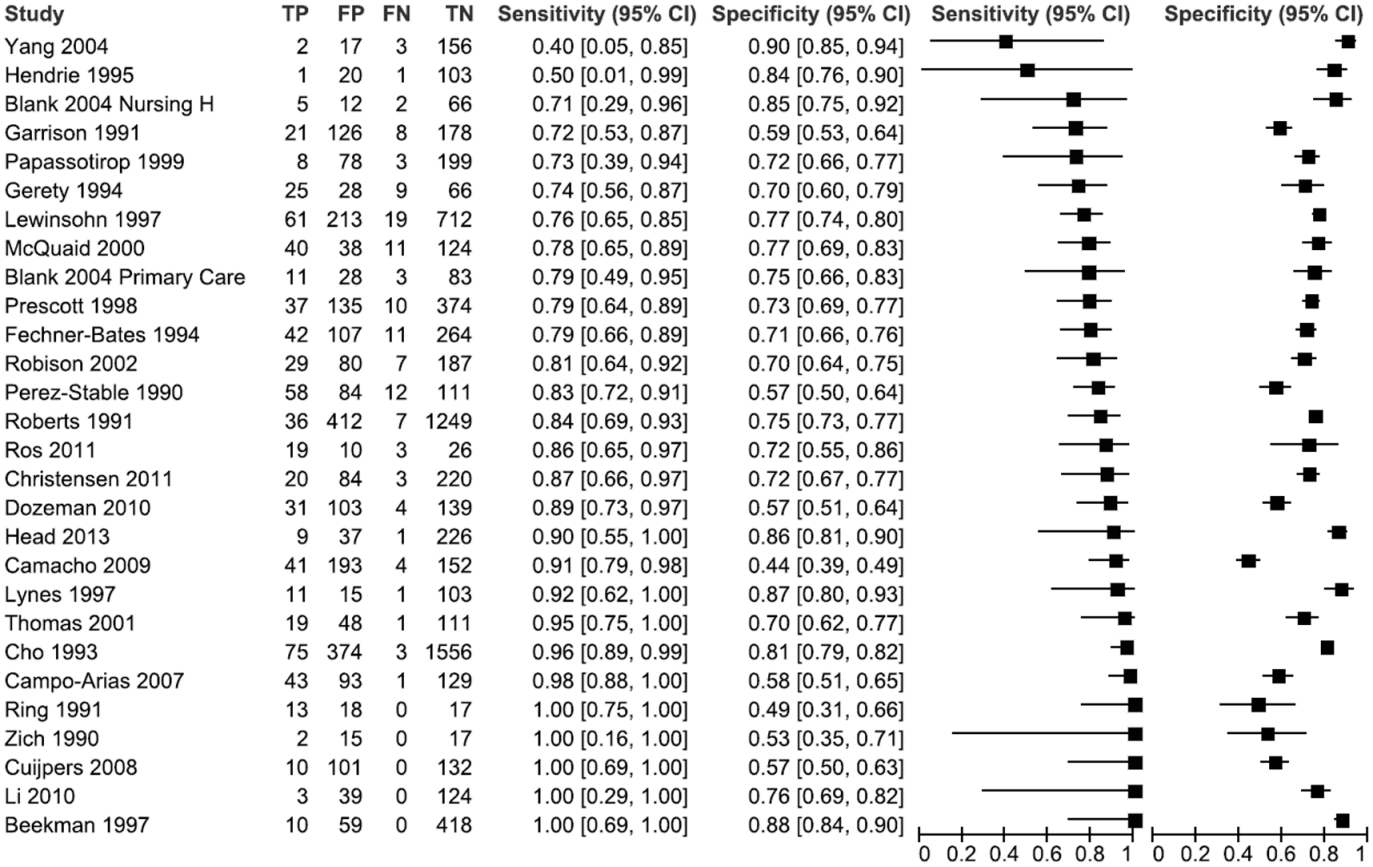
Coupled Forest plot of sensitivities and specificities of included studies (n = 28).

The median sensitivity was 0.85, with a range from 0.40 to 1.0. Specificities ranged from 0.44 to 0.90, with a median value of 0.72. Given that most of the studies used the same cut-off point, the V-form pattern showing threshold-like relationships, where specificity increased as sensitivity decreased, was not found. As expected, sensitivity had greater uncertainty, indicated by the confidence interval’s width, than specificity because the number of cases in all studies was generally lower than the number of non-cases (see coupled forest plot in Fig 2).

**Fig 3:**
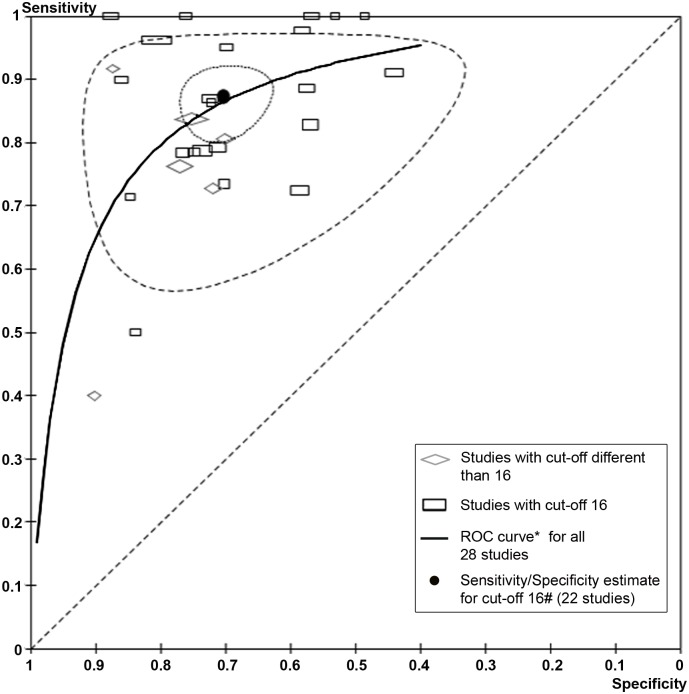
ROC scatter plot and ROC curve for all included studies (n = 28) and summary estimate for the subset of studies with cut-off point of 16 (n = 22). * Estimated with Rutter and Gatsonis hierarchical model; # Estimated with bivariate model.

Fig 3 presents the ROC scatter plot displaying the results of sensitivity and specificity for individual studies in the ROC space. It includes the 28 studies regardless of the cut-off used, and each study is plotted as a single sensitivity-specificity point. The size of the point depicts the precision of the estimate scaled according to the sample sizes, with height relating to the number of diseased (and hence precision of sensitivity) and width relating to the number of non-diseased (i.e. the precision of the specificity estimate). The scatter of point estimates showed that both indicators sensitivity and specificity presented similar variability. The SROC curve for all the 28 studies estimated from the Rutter and Gatsonis mixed effects model [26] was added to the graph. The black circle depicts the summary sensitivity and specificity point estimated with a bivariate model from the 22 studies that shared the same cut-off point of 16 (sensitivity = 0.87, specificity = 0.70). The corresponding 95% confidence region for the summary operating point and prediction region are also shown in the graph. The prediction region was large, indicating considerable between-study heterogeneity. Given that the sensitivity and specificity of a test varies as the positivity cut-off varies, a summary point could not be obtained for all the 28 studies. In the hierarchical model for the 28 studies, the shape parameter was not statistically significant, indicating that the SROC curve was symmetric and thus, the estimated DOR was constant across cut-offs. The estimated AUC was 0.87 following the calculation method described by Walter [28] under the assumption of symmetry. No clear pattern was observed with regard to the location of studies above or below the ROC curve depending on their size.

Pooled estimates of test accuracy, using bivariate models, were obtained for the cut-off points that were reported by at least 6 studies, i.e. 16, 20 and 22 (Table 2). For the cut-off point of 16, the estimated sensitivity from 22 studies was 0.87 (95% CI 0.82–0.91), and its specificity was 0.70 (95% CI 0.65–0.75). The positive likelihood ratio was LR+ = 2.94 (95% CI: 2.5–3.5), the negative likelihood ratio was LR- = 0.18 (95% CI: 0.1–0.3), and the resulting DOR was 16.2 (95% CI: 10.5–25.1). Twelve of the studies presented results for the cut-off point 20, with a pooled sensitivity of 0.83 (95% CI: 0.75–0.89), and specificity of 0.78 (95% CI: 0.71–0.83), and resulting DOR of 16.6 (95% CI: 9.7–28.5). The cut-off point of 22 was assessed in 7 of the studies, and the pooled estimate of sensitivity was 0.79 (95% CI: 0.69–0.85), specificity = 0.80 (95% CI: 0.75–0.85), with DOR = 14.7.

**Table 2:** Summary estimates of test accuracy measures at different cut-off points^#^.

	Cut-off point					
	≥16 (n = 22 studies)		≥20 (n = 12 studies)		≥22 (n = 7 studies)	
	Estimate	95% CI	Estimate	95% CI	Estimate	95% CI
Sensitivity	0.87	(0.82, 0.91)	0.83	(0.75, 0.89)	0.79	(0.69, 0.85)
Specificity	0.70	(0.65, 0.75)	0.78	(0.71, 0.83)	0.80	(0.75, 0.85)
Positive Likelihood ratio (LR+)	2.94	(2.46, 3.51)	3.69	(2.83, 4.80)	3.94	(2.92, 5.30)
Negative Likelihood ratio (LR-)	0.18	(0.13, 0.25)	0.22	(0.15, 0.33)	0.27	(0.18, 0.40)
Diagnostic Odds Ratio (DOR)	16.24	(10.49, 25.10)	16.64	(9.71, 28.51)	14.68	(7.71, 27.92)

^#^ estimated with bivariate model

None of the variables assessed as possible sources of heterogeneity were found to be statistically significant. Only the age covariate was close to statistical significance, but did not reach the nominal alpha level (p = 0.053). The graphical representation of the studies according to this variable seems to indicate lower diagnostic accuracy among the younger age group (see S2 Fig).

## Discussion

The CES-D was originally developed to measure depressive symptomatology in general population epidemiological studies [14], although it has also been extensively used in different settings both as a case-finding measure for depression and as a stand-alone diagnostic instrument. However, to date no effort has been made to obtain generalizable evidence through meta-analysis on the performance of the CES-D for these purposes in either the general population or in the primary care setting.

To our knowledge, this is the first study that presents pooled summary estimates of the indicators of accuracy for the CES-D scale at selected cut-off points, obtained using recommended hierarchical meta-analytic methods for synthesizing the results [58]. This study has considered the quality of the studies in a standardized way and it has analyzed possible sources of heterogeneity, to investigate whether the observed test accuracy varies between studies according to characteristics associated with their settings, participants or methodology.

Our systematic review has identified a total of 28 studies that show that the CES-D has acceptable screening accuracy for detecting major depression, and no significant differences between general population and primary care were found. However, at the most frequently recommended cut-off point of 16, its sensitivity is high at the expense of a moderate specificity. Our meta-analyses show that the cut-off point of 20 yields a more adequate trade-off between sensitivity and specificity, with higher specificity and lower sensitivity when compared to the cut-off point of 16.

### Limitations

Results presented here should be interpreted taking into account some limitations of our study. First, the results obtained for different cut-off points were based on a different number of studies and most of them presented results for only one, usually the recommended cut-off point of 16. Thus, evidence for other cut-off points is more limited. Of especial concern are the six studies that present results only for the optimal cut-off points within the study, an approach that might over-estimate test performance when compared to those studies using predetermined cut-off points, due to model over fitting [20]. Although, in our assessment of heterogeneity, no statistically significant differences in diagnostic accuracy were found between the two types of studies, our results might be biased towards a better performance of the CES-D in case detection. Second, we studied the CES-D only in the populations for which it was originally devised: general population and community surveys. The CES-D is also extensively used in clinical population studies which we did not include, given their heterogeneity in terms of conditions and designs. Thus their role as a source of heterogeneity remains to be evaluated. Third, prevalence and impairment in the 4 studies from residential settings, encompassing nursing homes [32;41], residential homes [38], and community centres for older adults [54] may differ from non-institutionalized elderly individuals in the general population or primary care. However, when we carried out a sensitivity analysis excluding these studies, the resulting sROC did not differ from that of the overall sample. Finally, it must be taken into account that this review assesses the properties of the instrument for major depression. Accuracy for other disorders could not be obtained due to inclusion criteria, which focused on major depression, and to the diversity and heterogeneity of the disorders or disorder categories other than depression evaluated in the included studies.

### Comparison with other studies

Several literature syntheses on performance of case-finding instruments for the identification of depression, including the CES-D, have been published previously [13;59;60]. Williams et al [13;59] evaluated the CES-D and 15 other case-finding instruments and reported an adequate performance in primary care, with a median sensitivity for all studies of 85% and a median specificity of 74%. None of the instruments showed superior performance characteristics. Among the several instruments reviewed by Watson et al. [60] for detection of late-life depression in primary care, the CES-D, the Geriatric Depression Scale (GDS), and the SelfCARE(D) presented similar accuracy, with sensitivities ranging from 74% to 100%, and specificities ranging from 53% to 98%. However, no meta-analytical methods were applied to synthesize the results, probably due to the low number of studies available for each of the instruments.

Our results for the CES-D are within the range of those described for the GHQ-12 [61], another symptom-based psychopathology scale that is commonly used in general population epidemiological surveys. This finding is remarkable as the GHQ-12 deliberately includes less specific mental distress symptoms, which are not exclusive of depression. In their review of validation studies using the GHQ-12 in primary care or community samples to evaluate its screening ability for common mental disorders, Goldberg et al. [61] reported sensitivity values ranging from 0.67 to 0.93 (median = 0.84) and specificity values from 0.59 to 0.91 (median = 0.79). In the same study, results were presented from primary care samples obtained in 15 centres around the world, showing areas under the curve ranging from 0.83 to 0.95 [61].

Perhaps the CES-D can be more fairly compared with the depression module of the Patient Health Questionnaire (PHQ-9), which has become increasingly popular over the past decade for detecting major depressive disorders in various clinical settings. The PHQ-9 takes account of the presence and severity of depression symptoms. A systematic review of the diagnostic accuracy of the PHQ-9 [62] including 14 studies reported a sensitivity of 0.80 (95% CI: 0.71–0.87) and a specificity of 0.92 (95% CI: 0.88–0.95). These values represent a positive likelihood ratio (LR) greater than 10 (LR+ = 10.12, 95% CI: 6.52–15.67), which has been claimed to generate large and often conclusive changes from pre-test to post-test probability [63]. The LRs obtained in our study for any of the cut-off points evaluated for the CES-D are considerably lower, between 3 and 4, values considered to generate small (but sometimes important) changes in post-test probability [63]. According to the results obtained here, for the commonly used cut-off point of 16, the CES-D scale would correctly identify 87% of the individuals with depression, while 30% of non-cases would be identified incorrectly as having the disorder (a positive LR of 2.94). With a cut-off point of 20, the CES-D scale would correctly identify 83% of the individuals with depression, while the percentage of non-cases that would be classified with the mental disorder would decrease to 22% (a positive LR of 3.7). Considering a maximum value of 10% for the12-month prevalence of depression in the general population [4], the observed LRs for the CES-D would represent a post-test probability in the range of 21% to 35% [64], while it would be substantially larger for the PHQ-9 (53%).

### Comparison of cut-off points

Selecting a cut-off point represents a trade-off between sensitivity and specificity and its appropriateness depends primarily upon the purpose of the instrument in a given study. When the CES-D is intended to be used in epidemiological studies to evaluate the relationships between depressive symptomatology and other variables across population subgroups, the cut-off point that provides an adequate tradeoff between sensitivity and specificity is recommended. On the other hand, when using CES-D as a case-finding instrument for identifying patients with clinically significant symptoms requiring additional evaluation, maximizing sensitivity should be of priority interest in order to minimize missed cases. However, in situations where medical and psychiatric resources are limited, it is also important to minimize the false-positive rate in order to reduce the burden of additional assessment for a final diagnosis. For example, if 10,000 individuals were screened, and assuming a 10% prevalence of depression, 1,000 individuals would be expected to have depression. Using the cut-off point of 16, 870 cases would be detected, whereas the cut-off of 20 would only detect 830 individuals (4.6% less). Concurrently, the number of individuals that would be wrongly classified as probably depressed and requiring additional assessment, that would end up being negative, would decrease from 2,700 for the cut-point 16 to 1,980 for the cut-point 20 (a 27% reduction). Consequently with the results obtained, the use of the CES-D as an isolated diagnostic tool is not recommended given the low positive likelihood ratio observed. Moreover, researchers considering the use of the CES-D as a diagnostic tool should bear in mind that its balance between sensitivity and specificity is inadequate for this objective, regardless of the cut-off point. Studies using the CES-D for diagnostic purposes should verify those diagnoses by conducting further assessment.

Notice that the incremental validity across different thresholds would be more adequately assessed in study designs obtaining accuracy results for a complete set of cut-off points within the same sample. Thus, there is need for more studies presenting paired results for the whole set of cut-off points.

### Sources of heterogeneity

The assessment of the quality of primary studies as well as other possible causes of between-study heterogeneity is important to ensure that inferences drawn from the review are appropriate [65]. We decided not to restrict the inclusion of the studies selected based on the results of the QUADAS tool. Instead, all relevant evidence was evaluated and the possible sources of heterogeneity were assessed by including them in meta-regression analyses to investigate the association of each of these sources with the estimated accuracy, as recommended previously [66]. Among the possible sources of heterogeneity evaluated, none of the variables under consideration were found to be statistically significant. The only variable that came close to being statistically significant was age, but it still failed to meet the nominal alpha level. This result is certainly a power issue, as the number of studies was not large enough. Given the restricted sample size, the only possible recommendation would be to promote new studies about diagnostic accuracy. In line with our results, other studies were unable to detect a significant effect of partial verification (i.e. selectively including patients with positive test results) on the DOR [67;68]. However, a previous study [66] showed that partial verification, would introduce bias because patients with false-negative test results would remain undetected and sensitivity would be overestimated. Our results indicate that this is not the case, although a high proportion of studies that performed partial verification actually did correct their results for the oversampling of positive test respondents. An additional comparison of the accuracy of studies that did not account for partial verification in the analysis with the rest of the studies did not show statistical significance either.

Contrary to expectations, some of the studies using a linguistic adaptation of the CES-D showed sensitivities and specificities well over those of studies using the original English version. However, adaptation was not found to be a significant source of heterogeneity in the analysis of covariates. Given the small number of studies involved, the potential influence of the language and cultural adaptation of the questionnaire requires further research. In general, the p-values of the variables under consideration were not close to the alpha level and, with models of this type, it is difficult to translate model parameters into a meaningful effect size that helps to carry out a substantive interpretation of the accuracy by covariate subgroups.

### Recommendations for future research

Our review uncovered that the design and methodology was insufficiently or inadequately reported in some of the studies. Authors should be encouraged to follow published guidelines, the Standards for Reporting of Diagnostic Accuracy (STARD) [69], in order to improve the accuracy and completeness of the reports of studies of diagnostic accuracy, which will allow a better assessment of the risk of bias in future studies.

An important issue that has not been addressed here is the fact that the categorical status of psychiatric diagnoses is controversial. As the continuum hypothesis of psychopathology gains ground in psychiatric research, more epidemiological studies exploring the relationships between severity and the diagnostic status of psychiatric syndromes will be necessary.

## Conclusions

In conclusion, the accuracy of the CES-D is acceptable for its use as a first-stage screener to target respondents with depressive symptoms for more in-depth clinical assessment, but its use as a stand-alone measure for diagnostic purposes is not recommended. A cut-off point of 20 seems to provide a better trade-off between sensitivity and specificity than the typically recommended value of 16, and is more advisable in certain applications where the resources are limited, representing a significant reduction on the burden of additional assessment without a great loss of positive cases.

## Supporting Information

S1 Dataset Dataset for meta-analysis. (XLS)

S1 Fig Methodological quality graph: review authors' judgments about each methodological quality item from QUADAS presented as percentages across all included studies. (TIF)

S2 Fig ROC Scatter plot of included studies by age group. (TIF)

S1 PRISMA Checklist PRISMA Checklist. (PDF)

S1 Table Search strategy conducted in PubMed. (PDF)
