# Supplementary figures and images for: Screening for Depression in the General Population with the Center for Epidemiologic Studies Depression (CES-D): A Systematic Review with Meta-Analysis
Source: PLoS One. 2016 May 16;11(5):e0155431. doi: 10.1371/journal.pone.0155431 (PMC4868329; doi:10.1371/journal.pone.0155431)

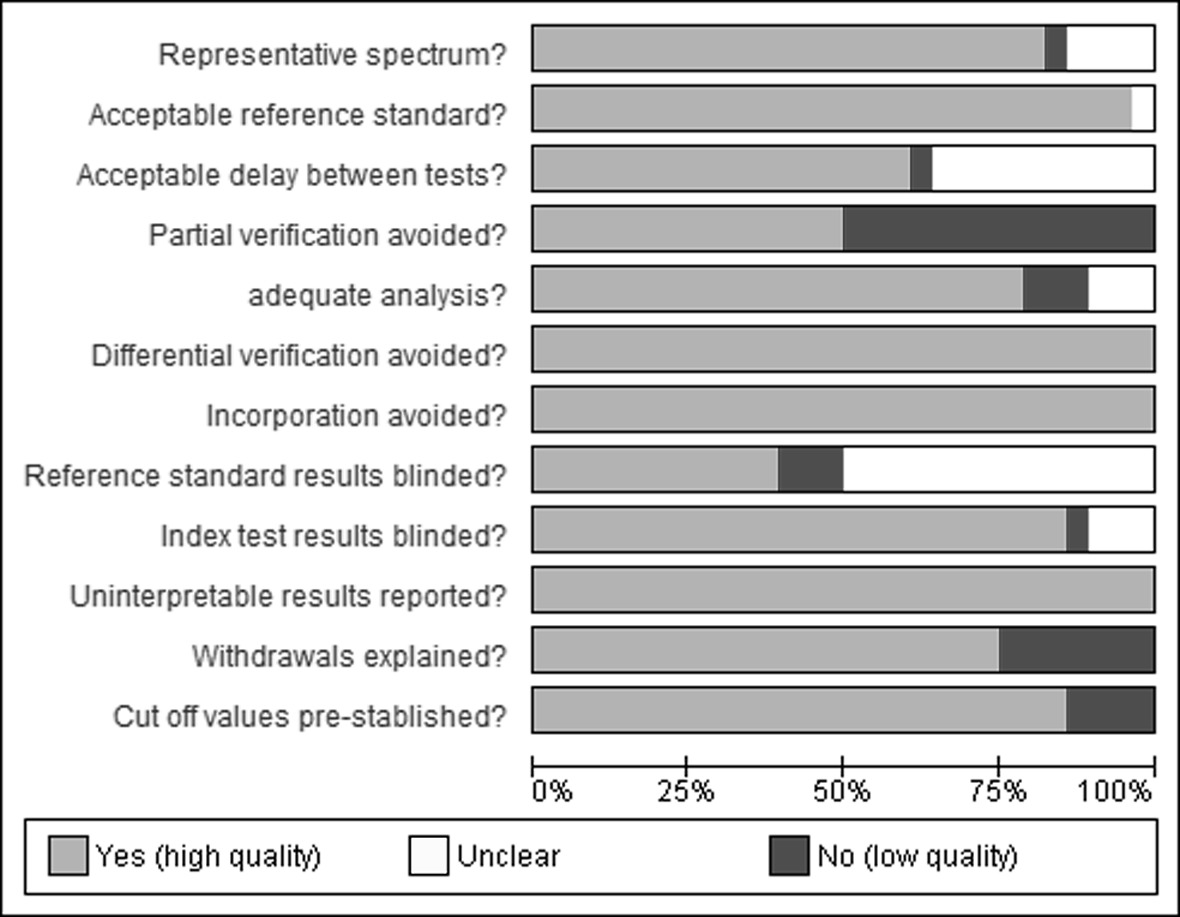

Supplement: S1 Fig — (TIF) [file pone.0155431.s002.tif]

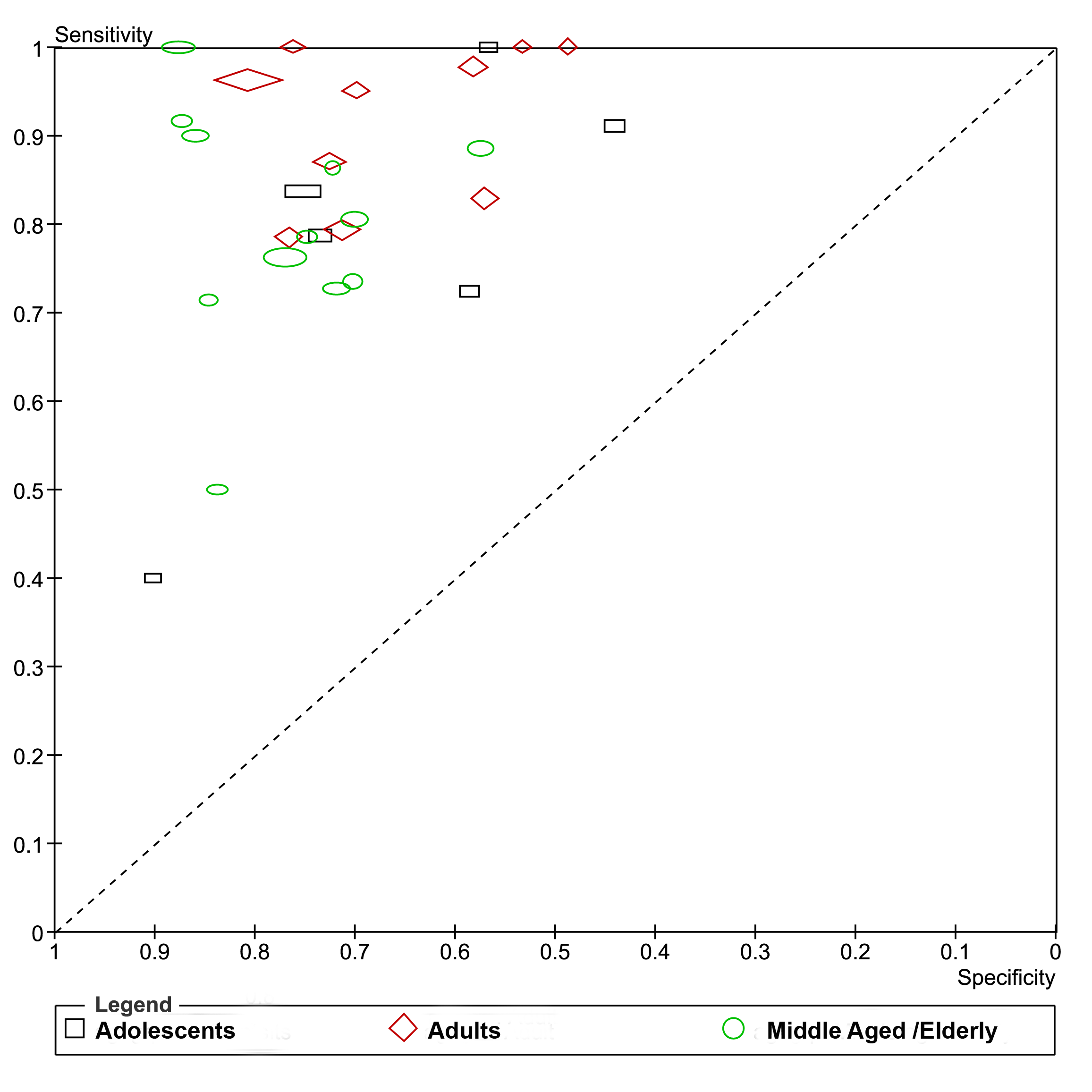

Supplement: S2 Fig — (TIF) [file pone.0155431.s003.tif]
