# Supplementary material for: Screening for Depression in the General Population with the Center for Epidemiologic Studies Depression (CES-D): A Systematic Review with Meta-Analysis
Source: PLoS One. 2016 May 16;11(5):e0155431. doi: 10.1371/journal.pone.0155431 (PMC4868329; doi:10.1371/journal.pone.0155431)
Supplement: S1 Table — (PDF) [file pone.0155431.s005.pdf]

S1 Table. Search strategy conducted in PubMed

Search #	Search details
**COMPONENT 1. MENTAL DISORDERS:**	
#1	Depressive Disorder[Mesh:noexp]
#2	depression
#3	Anxiety[Mesh:noexp]
#4	Anxiety disorders[Mesh:noexp]
#5	Mental disorders[Mesh:noexp]
#6	depressive*[Title/Abstract] OR depression*[Title/Abstract] OR anxiety*[Title/Abstract] OR mental disorder*[Title/Abstract]
#8	(#1 OR #2 OR #3 OR #4 OR #5 OR #6)
**COMPONENT 2. SCREENING INSTRUMENTS:**	
#9	"general health questionnaire"[Title/Abstract]
#10	"Center for Epidemiologic Studies Depression Scale"[Title/Abstract]
#11	"Epidemiologic Studies Depression Scale"[Title/Abstract]
#12	"GHQ-12"[Title/Abstract] OR "GHQ12"[Title/Abstract]
#13	"CES-D"[Title/Abstract] OR "CESD"[Title/Abstract]
#14	screen*[Title/Abstract]
#15	Mass screening[Mesh:noexp]
#16	(#9 OR #10 OR #11 OR #12 OR #13 OR #14 OR #15)

**COMPONENT 3. DIAGNOSTIC ACCURACY :**	
#17	(Curve, ROC) OR (Curves, ROC) OR (Analysis, ROC) OR (Analyses, ROC) OR (Receiver Operating Characteristic) OR (Receiver Operating Characteristics)
#18	Sensitivity AND Specificity
#19	Validation*
#20	#17 OR #18 OR #19
**LANGUAGE LIMIT:**	
#21	"english"[Language] OR "spanish"[Language]
#22	#8 AND #16 AND #20 AND #21
